# Intracellular dry mass density increases under growth-induced pressure

**DOI:** 10.12688/openreseurope.18557.2

**Published:** 2024-12-19

**Authors:** Hyojun Kim, Baptiste Alric, Nolan Chan, Julien Roul, Morgan Delarue

**Affiliations:** 1 LAAS-CNRS, University of Toulouse, Toulouse, France; 2 Institute of Industrial Science, Tokyo University, Tokyo, Japan; 3 Phasics, Bâtiment Mercury I, Espace Technologique, Route de l’Orme des Merisiers, St. Aubin, France

**Keywords:** macromolecular crowding, microfluidic, growth-induced pressure, quantitative phase imaging, refractive index, dry mass density

## Abstract

Cells that proliferate in confined environments develop mechanical compressive stress, referred to as growth-induced pressure, which inhibits growth and division across various organisms. Recent studies have shown that in these confined spaces, the diffusivity of intracellular nanoparticles decreases. However, the physical mechanisms behind this reduction remain unclear. In this study, we use quantitative phase imaging to measure the refractive index and dry mass density of *Saccharomyces cerevisiae* cells proliferating under confinement in a microfluidic bioreactor. Our results indicate that the observed decrease in diffusivity could be attributed to the intracellular accumulation of macromolecules. Furthermore, the linear scaling between cell content and growth-induced pressure suggests that the concentrations of macromolecules and osmolytes are maintained proportionally under such pressure in *S. cerevisiae*.

## Plain language summary

Cell proliferation in confined environments leads to the buildup of mechanical pressure. Mechanical pressure has been associated with the decreased motion of intracellular nanoparticles, but the physical basis for this slowdown has not been revealed. In this study, we measure the change in dry mass density of budding yeast growing in a confining microfluidic chamber using quantitative phase imaging. The dry mass density of cells increases linearly with pressure, which can be explained by continued mass accumulation in constrained cell volume. Our results suggest that the accumulation of mass beyond cellular homeostasis may be the physical driver of the restricted growth of cells in a confined space.

## Introduction

Most cells live in spatially-confined environments. Spatial confinement can be found everywhere in the living, from roots sprouting into the porous soil to tumors growing in the body of an organ, all the way to microbes growing in confining biofilms. Living cells proliferating in confined spaces eventually build up mechanical compressive stress exerted onto themselves and their surroundings. This growth-induced pressure (GIP) has the potential to decrease cell growth in all kingdoms of the living, including bacteria ^1– 3^, fungi ^4, 5^, plants ^6, 7^ or mammals ^8– 10^. It has recently been shown in the budding yeast *Saccharomyces cerevisiae* ( *S. cerevisiae for short*) ^11^, in bacteria ^3^ and in mammalian cells that GIP is accompanied by a decrease in the diffusion of genetically-encoded tracer nanoparticles. This decreased diffusion has been attributed to an increase in intracellular density: continued biosynthesis with limited cell volume increase would lead to increased biomass within the cytoplasm. However, this decrease in diffusion could also be attributed to other effects, such as a decrease in biochemical activity which is known to fluidize the cytoplasm ^12, 13^, or an acidification of the cell milieu which can lead to decreased fluidity ^14^. Hence, it remains to be shown that confined growth could lead to the accumulation of macromolecules, and, consequently, to an increase in intracellular density.

The dry mass density of a living cell can be a direct proxy of macromolecular concentration. Numerous methods exist to measure the dry mass density of living cells, such as suspended microchannel resonator, measuring single cells’ buoyancy suspended in media ^15, 16^, or cryoelectron tomography, which requires cell fixation ^17, 18^. However, these methods necessitate to recover the sample or are not compatible with spatial confinement. Indeed, to develop GIP, cells are proliferating inside a confining microfluidic chamber and become densely packed ^4, 8^. Cells cannot be recovered from the system, which is inherently incompatible with suspended microchannel resonator or cryoelectron tomography, limiting the measurement of dry mass density to optical methods. As such, quantitative phase imaging (QPI) is an alternative technique that can non-invasively measure the dry mass density of optically transparent biological cells or tissues ^19, 20^.

QPI can quantify the refractive index (RI) distribution of a biological sample by detecting the phase difference of light passing through the sample with the surrounding media. This measured RI is directly proportional to the dry mass density of a biological sample ^21^. In this paper, we investigated the changes in the dry mass density of the budding yeast *S. cerevisiae* growing within a confined space using QPI, and examined how it correlated with the concentration of a biological fluorophore, serving as a proxy for protein concentration.

## Methods

### Cell culture conditions and strain

*S. cerevisiae* cells were grown and maintained on a Synthetic Complete (DCS0019, Formedium) + 2% dextrose (SCD) media agar Petri dishes. Next, a single colony was inoculated in a fresh SCD liquid media and incubated with orbital shaking at 200 rpm at 30°C overnight. Exponentially growing culture at OD = 0.3 was then loaded into the microfluidic chamber. Green fluorescent protein (GFP) was expressed from a *P _HIS3_-GFP* construction in a W303 background (gift from Liam Holt’s lab). *HIS3* promoter is constitutively active, such that GFP is constantly produced by the cell. Growth rate of the GFP strain is similar to the corresponding wild-type strain (data not shown).

### Microfabrication of microfluidic devices

The mold with two layers of different heights is fabricated in a cleanroom at LAAS-CNRS using classical photolithography with negative photoresists. The first layer at a height of 0.8 μm defining the culture media channels was prepared with 2mL of negative photoresist (SU8 3000.5, Kayaku Advanced Materials), and the second layer at a height of 9.2 μm defining the cell growth chamber and the main cell-loading channel was prepared with 2mL of another negative photoresist (HARE-SQ10, Kemlab). After the lithography process, perfluorodecyltrichlorosilane was supplied in 100 sccm for 10 seconds and grafted with O _2_ plasma under 40 Torr of pressure onto the wafer surface using an SPD (Memsstar Technology) machine to improve the hydrophobicity and non-stiction. Polydimethylsiloxane (PDMS) elastomer was prepared by mixing the base and curing agent in a 10:1 ratio, poured onto the mold, and cured overnight at 60°C. Both PDMS and a glass coverslip were activated with oxygen plasma treatment (Diener PICO; gas, oxygen; pressure, 0.3 mbar; power, 100%; activation, 20 s), and bonded to each other immediately after surface activation. The PDMS/glass chip was then baked for ≥5 h at 60°C.

### Microfluidic device operation

The cell suspension was introduced to the chambers by hand using a syringe – we typically fill 10–20% of the chamber within 1 minute. We next injected the SCD media through the main inlet channel using a Fluigent MFCS™ pressure control system, with a pressure of 1 bar. During the cell incubation and imaging, the pressure at the culture media inlet was maintained at ~1 bar. The chips were placed in a microscope environmental chamber at 30°C (TempControl-37, Leica Microsystems). To prevent nutrient depletion and exchange media, chambers were supplied with nutrients through microchannels on both sides. GIP was measured through the deformation of the PDMS elastic wall. Note that PDMS is permeable to gases, such as oxygen.

### Refractive index measurement of bulk liquid/solid samples

Liquid and solid samples were measured using an Abbe refractometer (2WAJ, OPL) with white light at 30°C. Culture media (SCD) and distilled water samples were measured in spread, like a thin film, between the main and secondary prisms. A cured PDMS sample was prepared into a film of about 500 μm thickness and was carefully placed between the two prisms' surfaces without any air bubbles. The RI of each sample was consistent for three independent measurements, and errors were not stated because the variances were smaller than the refractometer's measurement resolution (10 ^-4^).

### Bright field and fluorescent microscopy

All imaging acquisition in this study was conducted on an inverted microscope (DMi8, Leica Microsystems). The lateral deformation of PDMS chambers was measured to infer growth-induced pressure through bright-field microscopy simultaneously with the QPI. The relationship between pressure and chamber deformation was calibrated as done in previous work ^10, 11^, giving a value of 8.2 μm.MPa ^–1^.

To measure GFP accumulation, cytosolic GFP expressed from *HIS3* promoter were measured by fluorescent z-stacking (0.5 μm interval) with a spinning-disk confocal scanner unit (CSU-X1, Yokogawa). We acquired z-stack of the first cell layer of each chamber using the same way of measurement for the chamber height with ET525/50 nm red emission filter, a dichroic mirror (ZT405/488/561/638rpc, Chroma) and Hamamatsu scientific complementary metal–oxide–semiconductor camera (ORCA-Flash4.0 v3, Hamamatsu Photonics). In the same way, we imaged the cells without any GFP labeling to subtract the cellular autofluorescence which can be detected from GFP channels. We equally subtracted the fluorescence background. The fluorescence intensity was homogenous across the chamber, suggesting that there were no large variations of intercellular GFP expression. The GFP expression of each cell was calculated by summing of pixel values across all z-stack slice of an chamber in ImageJ/Fiji ^22^.

### Refractive index and dry mass density measurement of cells with QPI

For transmission QPI, the microscope was equipped with conventional LED Köhler transillumination and a condenser of maximum numerical aperture NA = 0.55. To acquire QPI data, we imaged the samples with a 20x 0.8 NA objective (Leica) and a quadriwave lateral shearing interferometry (QWLSI) system (SID4 sc8, Phasics) mounted on the microscope’s lateral camera port. The QWLSI measures the local phase shift, also called optical path difference (OPD), introduced by a specimen placed under a microscope. Depending on the sample's thickness and the difference of sample's refractive index from the background material, the OPD was depicted in grayscale on the image, the so-called phase image.

To estimate RI of cells (n _cell_) on a culture plate, every cell was segmented from a phase image, which set the culture media as a background baseline, using Otsu algorithm method by CellProfiler ^23^. The n _cell_ is estimated by the relation, n _cell_ = n _background_ + OPD/d _cell_, where the OPD is difference between cell and background and d _cell_ is the height of the cell. Since cells were assumed to be prolate ellipsoids, the d _cell_ was taken as the length of the minor axis of the cell in the phase image. The corresponding OPD was taken as the brightness of the upper 5% of the intensity distribution within the cell from the phase image in which the background was subtracted.

To measure the average RI of cells within a microfluidic chamber, the averaged OPD value along the chamber area was measured, comparing to surrounding PDMS. To minimize the influence of local and variable OPD gradients occurring in phase images of cells within the PDMS microfluidics chip, we took the average value of the PDMS area in all directions surrounding the cell sample as the background baseline of the measurement. The chamber height under pressure was determined by the OPD of the calibrated culture medium of chambers expanded by predefined hydraulic pressure: d _chamber _= OPD/(n _PDMS_-n _medium_). Measurement of chamber’s height at GIP = 0 MPa perfectly matched the measurement of the SU8 mold measured with a profilometer. For GIP > 0 MPa, the cellular RI was measured in comparison to PDMS: n _cell_(P) = n _PDMS_ + OPD(P)/d _chamber_(P).

The dry mass density of a yeast cell is directly calculated from the mean RI value since the RI in biological samples is linearly proportional to the dry mass density inside cells as n _cell_ = n _media_ + αρ, where n _cell_ is the average RI of each cell, n _media_ is the RI of the surrounding media which was obtained with Abbe refractometer (n _media_ = 1.336), α is an RI increment (α = 0.190 ml/g for proteins and nucleic acids ^19, 21^), and ρ is the dry mass density inside cells.

### Data and statistical analysis

All image analysis was performed using ImageJ (National Institutes of Health, US). Linear fitting was performed using the ordinary least squares model provided by the Statsmodels library in Python ^24^, while histograms and kernel density estimations were plotted using the histplot function from the Seaborn library ^25^.

## Results

### Refractive index and dry mass density of *S. cerevisiae* determined with QPI

**Figure 1.**
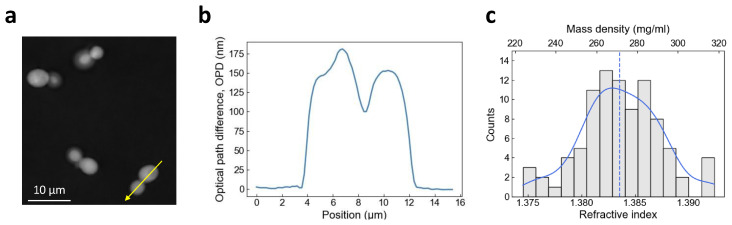
Measurement of cellular refractive index and dry mass density using QPI. ( **a**) Optical path difference (OPD) image of budding yeast cells in SCD medium. ( **b**) Profile plot drawn from ( **a**) following the yellow arrow line. ( **c**) Histogram of Refractive index (RI) and dry mass density measurements of budding yeast cells grown at 30°C in SCD medium. 91 cells were analyzed in this histogram. A kernel density estimation was plotted (blue) with the median value (dashed line).

We measured the RI and dry mass density of living yeast *S. cerevisiae* on a culture plate using QPI, which measures the phase difference between a sample and a reference in the image. Phase images displayed the optical path difference (OPD) between cells and the surrounding culture media in grayscale ( Figure 1a). The OPD was the difference in RI of a sample with regard to a reference (here, the culture media), times the height of the sample. The pixel value along the cross section of a cell was related to the physical thickness and the local RI of different parts of living yeasts, in which we could for instance identify the vacuole (small initial bump) and the bud neck in between the mother part and the bud part of the cell ( Figure 1b). We measured the maximum OPD of each cell with respect to the culture media to be 180 ± 20 nm. We estimated the average cellular height to be 3.94 ± 0.50 μm (see Materials and Methods). Figure 1c displays the histogram of the RI measured from single cells, from which we determined the mean RI and dry mass density of an asynchronous population of yeast *S. cerevisiae*, n _cell_ = 1.384 ± 0.004 and ρ _cell_ = 271 ± 19 mg/ml.

### Increased intracellular density under GIP

**Figure 2.**
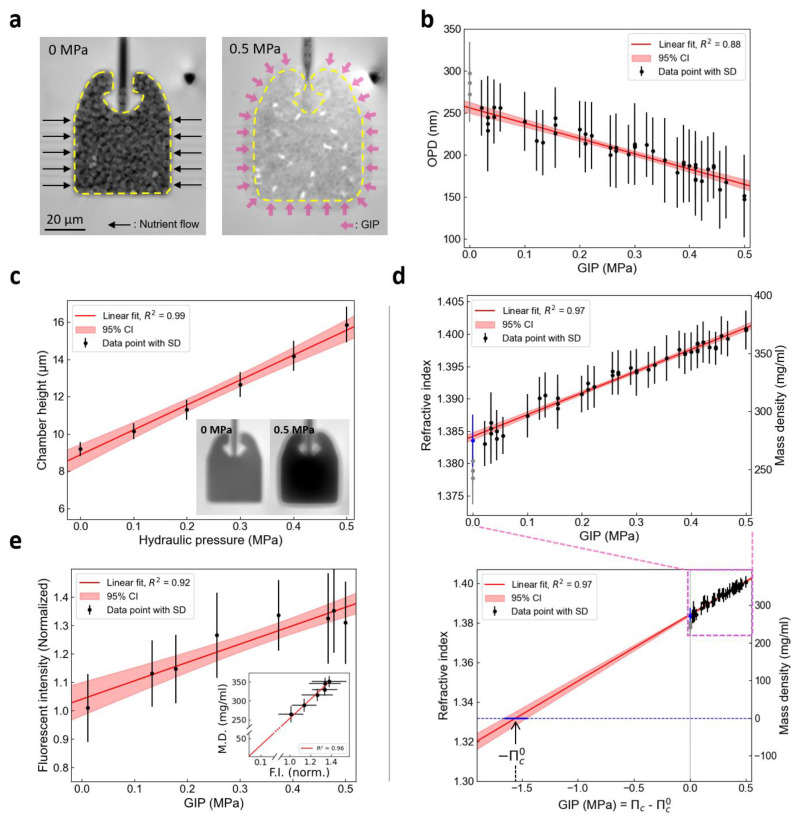
Intracellular dry mass density of budding yeast increases linearly with growth-induced pressure. ( **a**) OPD images of budding yeast cells growing within the confining space of a PDMS microfluidic chamber. Cells do not experience growth-inducing pressure until their growth fills the entire volume within the chamber (left, GIP = 0 MPa). After, confined growth leads to the build-up of growth-induced pressure (GIP), deforming the elastic chamber, where the expanded boundary is labeled with a yellow dashed line (right, GIP = 0.46 MPa). ( **b**) Change in OPD of cells under GIP measured relative to surrounding PDMS (n = 45 over 3 independent replicates). ( **c**) The effective height of the PDMS chamber linearly increased with the GIP level, which is proportional to the OPD between the chamber and surrounding PDMS (inset). ( **d**) The RI and dry mass density increase linearly as a function of GIP. The value estimated outside of the device previously is presented in a blue point. The measurements within the device at GIP = 0 are presented in grey, which were excluded from the regression as they are assumed to be underestimated. The nominal intracellular osmotic pressure ( $\Pi_{c}^{0}$ ) is estimated through linear extrapolation. An estimated $\Pi_{c}^{0}$ value is indicated. ( **e**) Fluorescence intensity (F.I.), corresponding to GFP expression level, linearly increases along GIP (each point is n ≥ 30 cells). Dry mass (D.M.) density is proportional to fluorescence intensity (inset). The values in the inset graph is denoted by mean ± standard deviation in both the x and y-axis direction.

We investigated the change in intracellular dry mass density as a function of growth-induced pressure (GIP). We used microfluidic elastic chambers to grow *S. cerevisiae* within a confined space to investigate the cellular RI under GIP ^11^. Figure 2a displays the confining chamber, and showcases the typical deformation upon GIP and the nutrient channels allowing cell feeding. After the cells filled the space through proliferation, they developed GIP by pushing against their neighbors and onto their surroundings, deforming the chamber.

We acquired phase images of cells in a chamber under GIP using QPI to examine cellular RI and observed on Figure 2b that the average OPD of the cells with respect to the surrounding PDMS decreased linearly with increasing pressure. As PDMS has a higher RI than the cells on average (n _PDMS_ = 1.405) the decrease in OPD implied that the RI of a cell approached the value of PDMS with increased GIP. The effective height of the deformed confining chamber was calibrated by measuring the OPD of the chamber filled with culture medium and pressurized by a measured hydraulic pressure. We observed a linear increase of the chamber height with respect to hydraulic pressure ( Figure 2c). This allowed us to extract, assuming that the chamber was fully filled with cells, the mean cellular RI as a function of GIP. We showed that RI increased roughly linearly with increased GIP under confinement ( Figure 2d).

We noted that the RI of cells without pressure in the chambers was underestimated (gray points in Figure 2d, compared to the blue point measured outside of the device). We attributed this underestimation to the fact that cells were not tightly packed in the chamber, lowering the effective RI due to culture medium at a non-negligeable volume fraction in the chamber.

### Proportional increase in GFP production with intracellular density under GIP

We measured the mean fluorescence intensity of a GFP continuously expressed from a *HIS3* promoter. We showed a linear increase of GFP concentration in the cell as a function of GIP ( Figure 2e). Interestingly, we observed that the increase in mass density was proportional to the increase in GFP concentration ( Figure 2e, inset), suggesting that the linearity between RI and protein concentration was kept in these conditions. Importantly, both the intracellular density and the fluorescence intensity extrapolated to 0, confirming the good proportionality of intracellular density and GFP amount. These results together demonstrated that the cellular biomass and GFP concentration increase roughly proportionally to GIP.

### Estimation of the nominal intracellular osmotic pressure

The intracellular RI is proportional to the dry mass of the cell. Figure 2d showed that mass and intracellular osmotic pressure increased proportionally under confined growth. We performed a linear extrapolation of the pressure to a RI matching the one of water at 30°C (n _water_ = 1.332). The points at GIP = 0 MPa were not considered (see discussion above), and we instead considered the RI of cells measured in culture medium (blue point). We assumed that when the cell would have the RI of water, it would be empty of its constituents, and its intracellular pressure would then be null, *Π _c_* = O MPa. When the RI would match the RI of water, it would thus correspond to a point where the cell would be “empty” of macromolecules. This GIP value, denoted $P_{n_{\text{water}}}=\Pi_{c}-\Pi_{c}^{0}$ , corresponds to the pressure difference between the intracellular osmotic pressure cell, *Π _c_*, and the nominal intracellular osmotic pressure, $\Pi_{c}^{0}$ . This value allowed us to estimate the nominal intracellular pressure of the cell: $\Pi_{c}^{0}=-P_{n_{\text{water}}}$ ( Figure 2d). We found in this case a nominal pressure of 1.55 MPa and the 95% confidence interval ranges from 1.46 to 1.66 MPa.

## Conclusion and discussion

In this study, the refractive index measured at the single cell level lied within the range of the RI measured for various yeast cells ^26– 30^. Moreover, we showed that the dry mass density under pressure can be estimated by quantitative phase microscopy, and increased with GIP. The previous measurement of intracellular density under GIP has only been estimated indirectly by tracking fluorescently-tagged tracer nanoparticles. It was assumed that the decrease in particle diffusion occurred due to the reduction of the free volume of the cytoplasm ^11^. However, the macromolecular diffusion within the cytoplasm, a highly heterogeneous active media, can be limited not only by the accumulation of macromolecules but also by a change in macromolecule size distribution ^31^, molecular electrostatic interactions ^32^, or active random force ^33^. Therefore, our results experimentally verify that the physical basis of the crowding-induced reduction in particle diffusion is compatible with an increase in dry mass density.

The previous theoretical model relating nanoparticle tracer diffusion with GIP assumed that the concentrations of osmolytes and macromolecules were produced proportionally ^10, 33^, but this was not confirmed experimentally. The linear relation between GIP and RI shown in our results indicated that the production ratio of macromolecules and osmolytes was kept constant under pressure in the yeast *S. cerevisiae* ( Figure 2d). The understanding of the relationship between growth and mass regulation is of primordial importance, as it is at the basis of intracellular density homeostasis ^3, 34^. Our results show that the molecular basis for density homeostasis seems to be unaltered under confined growth and increased density.

The nominal osmotic pressure of budding yeast estimated through linear extrapolation, 1.55 MPa, was slightly larger than the value obtained from the previous study's theoretical model, 0.95 MPa ^10^. This discrepancy could originate from the different methods and hypotheses used to evaluate this pressure. Here, we used an extrapolation assuming a linear trend, while the previous estimation was obtained from the nuclear volume decrease, under the assumption that the pressure inside the nucleus remained constant as the cytosolic pressure was increased. So far, we have no means of testing independently both hypotheses, but we noted that these values remained in the same order of magnitude.

## Abbreviation list

GIP: Growth-Induced Pressure

GFP: Green Fluorescent Protein

OPD: Optical Path Difference

PDMS: PolyDiMethylSiloxane

RI: Refractive Index

SCD: Synthetic Complete Dextrose

## Ethics and consent

Ethical approval and consent were not required.

## Data Availability

All acquired data and the codes developed in this work are freely available under the DOI: 10.5281/zenodo.1384264 here: https://zenodo.org/records/13842648 ^35^ Each excel file tab corresponds to the data plotted in the different sub-figures.
