# Supplementary material for: Intracellular dry mass density increases under growth-induced pressure
Source: Open Res Eur. 2024 Dec 19;4:231. Originally published 2024 Oct 21. [Version 2] doi: 10.12688/openreseurope.18557.2 (PMC11809470; doi:10.12688/openreseurope.18557.2)
Supplement: Peer review history

# Reviewer report: Reviewer response for version 2

**DOI:** 10.21956/openreseurope.20720.r49193

**Published:** 2025-01-22

**Authors:** Sara Mouton

**Affiliations:** 1 University of Groningen, Groningen, The Netherlands

In the current manuscript, Kim and colleagues provide experimental evidence for the increase of intracellular dry mass density due to increasing growth induced pressure. Previously, the laboratory has shown that yeast cells experiencing mechanical compressive stress exhibit a decreased motion of genetically encoded mesoscale nanoparticles. Here, the authors present evidence in favour of the explanation that the observed decreased diffusion rates are due to increased macromolecular crowding. The authors utilize a microfluidic chamber to trap and mechanically compress yeast cells, and use microscopy techniques to examine the relationship between buildup of mechanical force and increased refractive index, extrapolating increasing density of the intracellular mass. The findings are presented in a logical way and the data is clear.  suggest a few minor textual changes to improve the readability and reproducibility of the findings. In addition, I have some comments about Figure 2e and some suggestions for the clarification of methodology.

**Minor comments and textual changes:**

 1. " *Spatial confinement can be found everywhere in the living,...*" - this sentence needs rephrasing e.g. spatial confinement can be commonly found in living systems/all domains of life/etc.

 2. " *This growth-induced pressure (GIP) has the potential to decrease cell growth in all kingdoms of the living,...*" rephrase to all kingdoms of life/living organisms.

 3. "... *a decrease in biochemical activity which is known to fluidize the cytoplasm* ^*12, 13* "^. Both of these references look at motion of chromosome loci. While relevant references, I am not completely sure these studies show that reduced biochemical activity fluidizes the cytoplasm in particular. Perhaps Xie et al. 2024, *Mol Cell* will be a better reference here. 

 4. Before the sentence: " *Indeed, to develop GIP, cells are proliferating inside a confining microfluidic chamber and become densely packed*" there needs to be a sentence added to provide a transition from background information to the current experimental design. 

 5. " *primordial importance*" - Please rephrase. 

**Clarification of methods and data analysis/interpretation:**

 1. " *Green fluorescent protein (GFP) was expressed*

*from a P* *HIS3* *-GFP construction in a W303 background (gift from Liam Holt’s lab)"  - *If this strain was published previously, the name of the strain along with a reference is needed. If the strain is published in this paper for the first time then some details about the strain construction need to be included. 

 2. While the working principle of the microfluidic device is clear, some details about the timing are missing. Was there any incubation period before the cells were imaged inside the device? How long did the cells spend in the device? How was the endpoint of the experiment determined? These details will facilitate reproducibility of the results. 

 3. " *To measure GFP accumulation*, *cytosolic GFP expressed from HIS3 promoter were measured by fluorescent z-stacking*". This sentence and the whole paragraph can benefit from use of more precise terminology. Fluorescent z-stacking = fluorescence microscopy? Additionally, please include imaging settings, such as exposure time and percent transmission.

 Below I highlight some of the sentences in the paragraph that need further clarification: 

 - " *We acquired z-stack of the first cell layer of each chamber...*" I was under the impression that the cells are in a monolayer. I would be grateful if further clarification is provided here. 

 - " *We equally subtracted the fluorescence background*."- I am not sure what is the meaning of this sentence. 

 - " *The fluorescence intensity was homogenous across the chamber, suggesting that there were no large variations of intercellular GFP expression.*" Variations within individual cells or variations between cells? Indeed, between individual cells the fluorescence intensities seem to vary within a 25% margin when no pressure was applied. The use of the word "homogeneous" implies there could be a pattern in the fluorescence signal, while the rest of the sentence refers to fluorescence intensity. I suggest to omit the word homogeneous and discuss the intensity only e.g. " we did not observe high variation in the intensity of the fluorescence signal between individual cells (Fig 2e). 

 - " *The GFP expression of each cell was calculated by summing of pixel values across all z-stack slice of an chamber in ImageJ/Fiji ^22^.*". This sentence needs to be checked as well, both for English and precise terminology. 

 4. Regarding figure 2e: I was surprised by the high R ^2^ value of the linear fit. To make sure this was not a typographical error I replotted these values. I found the R ^2^ of the fit to be 0.89. I would be grateful if the authors can perform the analysis once more. The values are still very close, but possibly not completely correct. 

 5. The authors use the increase in GFP fluorescence intensity as a proxy for increasing protein concentration in the cells upon increased pressure. There are some issues with this method:  increased macromolecular crowding can quench the fluorescence of a variety of fluorescent proteins, including GFP (Morikawa et al. 2016, Scientific Reports). Hence, GFP fluorescence can serve as a proxy for increased protein concentration under the conditions presented here, but it is possible that the effects at higher densities are underestimated. Additionally, despite the high R ^2^ values, I am not sure how appropriate it is to state that the GFP fluorescence has a linear dependence on the pressure and dry cell mass density. Perhaps the authors should comment on this in the results section or alter their analysis and statement.

Is the study design appropriate and does the work have academic merit?

Yes

Is the work clearly and accurately presented and does it cite the current literature?

Partly

If applicable, is the statistical analysis and its interpretation appropriate?

Partly

Are all the source data underlying the results available to ensure full reproducibility?

Yes

Are the conclusions drawn adequately supported by the results?

Yes

Are sufficient details of methods and analysis provided to allow replication by others?

Partly

Reviewer Expertise:

Macromolecular crowding, yeast cell biology, yeast replicative ageing, fluorescence microscopy, genetically encoded fluorescent biosensors.

I confirm that I have read this submission and believe that I have an appropriate level of expertise to confirm that it is of an acceptable scientific standard, however I have significant reservations, as outlined above.

## References

[1] : Dependence of fluorescent protein brightness on protein concentration in solution and enhancement of it. *Sci Rep* .2016;6: 10.1038/srep22342 22342 10.1038/srep22342 26956628 PMC4783657

[2] : Polysome collapse and RNA condensation fluidize the cytoplasm. *Mol Cell* .2024;84(14) : 10.1016/j.molcel.2024.06.024 2698-2716.e9 10.1016/j.molcel.2024.06.024 39059370 PMC11539954

# Reviewer report: Reviewer response for version 2

**DOI:** 10.21956/openreseurope.20720.r48877

**Published:** 2025-01-09

**Authors:** Anahit Shirvanyan

**Affiliations:** 1 Biochemistry, Microbiology and Biotechnology, Yerevan State University, Yerevan, Armenia

I would like to express my gratitude to the authors for their comprehensive and detailed responses to my comments, as well as for their revision of the manuscript. The integration of the proposed changes has substantially improved the readability and accessibility of the manuscript.

 There remain a few minor technical errors that could be addressed to further enhance the overall quality of the manuscript. However, even if the authors opt not to address these issues, the manuscript is now considerably clearer and possesses substantial scientific merit. Therefore, I recommend this manuscript for indexing.

Is the study design appropriate and does the work have academic merit?

Yes

Is the work clearly and accurately presented and does it cite the current literature?

Partly

If applicable, is the statistical analysis and its interpretation appropriate?

Partly

Are all the source data underlying the results available to ensure full reproducibility?

Yes

Are the conclusions drawn adequately supported by the results?

Partly

Are sufficient details of methods and analysis provided to allow replication by others?

Partly

Reviewer Expertise:

Yeast Stress Biology, Biochemistry, Physiology, Bioenergetics

I confirm that I have read this submission and believe that I have an appropriate level of expertise to confirm that it is of an acceptable scientific standard.

# Reviewer report: Reviewer response for version 2

**DOI:** 10.21956/openreseurope.20720.r49194

**Published:** 2025-01-03

**Authors:** Jack W Shepherd

**Affiliations:** 1 University of York, York, UK

In “Intracellular dry mass density increases under growth-induced pressure”, the authors Hyojun Kim *et al *tackle *S cerevisiae *growth under confinement, which leads to pressure on the growing cells from the static walls of their confining box. This growth induced pressure leads to changes in the living cells such as decreased diffusion in the cytoplasm. The work here presents a potential physical origin of this decreased diffusion: a higher “dry mass” in the cytoplasm leads to diffusion slower than that in an unconfined unpressured cell. The authors’ choice of techniques is appropriate and all presented data point towards their conclusions, and I believe this work makes a useful contribution to the *S cerevisiae *and molecular crowding literature. Before indexing, some clarifications around fluorescence microscopy methods and data analysis would make the piece even stronger.

 Specific feedback for the authors

 1. There are a few points in the Introduction to address: delete “in the living” in the second sentence, “kingdom of the living” (“kingdoms of life”? I might remove the middle of that sentence so that it reads “...growth in bacteria, fungi, plants, and mammals”), “necessitate to recover” (replace with “necessitate recovering”), “an acidification” should just be “acidification”. “… can be a direct proxy” – can you be stronger here and just say “is”? “suspended microchannel resonator” needs a reference.

 2. Please clarify the *S. cerevisiae *strain in the first sentence of the Methods – is this is also W303?

 3. Some quantities have no space between the number and unit, e.g. 2mL, while others do have a space – this should be standardised

 4. “GIP was measured through the deformation…” needs a reference or an explanation of how this works

 5. “Culture media (SCD) and distilled water samples were measured in spread, like a thin film, between the main and secondary prisms.” Needs clarifications

 6. The lateral deformation of the PDMS appears in “Brightfield and fluorescence microscopy”, somewhat out of place. Should this be in “Microfluidic device operation”?

 7. I think SCMOS is a well known enough abbreviation for use in microscopy methods

 8. How exactly was autofluorescence and background calculated? Did you use Otsu thresholding or some other automatic segmentation or did you draw circles around the cells? Were the resulting cell autofluorescence pixel intensity values then averaged, and the average taken from each pixel intensity value in the GFP expressing cells?

 9. “The GFP expression of each cell was calculated” – not quite. To be precise, the summed pixel intensity values was calculated to give a total integrated intensity per cell. This is related to the expression of GFP but one would need to know the intensity emitted by one GFP molecule to get an expression level - and this could be complicated by the changing physical conditions within the cell. Specific to crowding, see Figure 1b here: https://www.nature.com/articles/srep22342

 10. Same sentence – summation of pixel intensity values was done with Fiji/ImageJ – Otsu thresholding as mentioned later, or some other manual/automatic segmentation routine?

 11. Objective lens part number, condensing lens part number, and if applicable immersion medium (water, oil, air) should be mentioned.

 12. “… was taken as the upper 5% of the intensity distribution …” is 5% a standard threshold to use here? If so, a reference would be appropriate, otherwise some justification of its choice needs inserting.

 13. “… since the RI in biological samples is linearly proportional to the dry mass density within cells …” Perhaps a reference here to aid the general reader?

 14. Data and statistical analysis section: Least squares fitting – did you weight any data points, and did you take into account error bars for these fits? Histograms and KDE plots – how did you decide on histogram bin widths and kernel widths?

 15. Increased intracellular pressure under GIP: the first two sentences here feel like repetition of earlier text; I would rework the first paragraph to remove redundancy. Probably the references to Fig 2a could be moved into the second paragraph and the first paragraph deleted.

 16. “Observed in” rather than “observed on” Fig 2b

 17. I have some concerns about Figure 2e. At high GIP the mean points appear to deviate significantly from linearity – at a guess it looks like there is a peak around 0.3 MPa. Why is linearity assumed? Similarly in the inset, there is deviation in the top right hand of the graph. Referring to my point 9 above, is it possible that a significantly more crowded environment has led to decreased GFP emission intensity?

 18. Figure 2e: what are the fluorescence intensities normalised to? Looks like the first data point but please mention this in the figure caption explicitly.

 19. “Intracellular pressure would then be null..” I think here you may have inadvertently used a capital “o” O rather than zero 0.

 20. “primordial importance” – primary importance?

Is the study design appropriate and does the work have academic merit?

Yes

Is the work clearly and accurately presented and does it cite the current literature?

Yes

If applicable, is the statistical analysis and its interpretation appropriate?

Partly

Are all the source data underlying the results available to ensure full reproducibility?

Yes

Are the conclusions drawn adequately supported by the results?

Yes

Are sufficient details of methods and analysis provided to allow replication by others?

Partly

Reviewer Expertise:

molecular crowding, fluorescence microscopy, S cerevisiae

I confirm that I have read this submission and believe that I have an appropriate level of expertise to confirm that it is of an acceptable scientific standard, however I have significant reservations, as outlined above.

## References

[1] : Dependence of fluorescent protein brightness on protein concentration in solution and enhancement of it. *Sci Rep* .2016;6: 10.1038/srep22342 22342 10.1038/srep22342 26956628 PMC4783657

# Reviewer report: Reviewer response for version 2

**DOI:** 10.21956/openreseurope.20720.r49192

**Published:** 2025-01-02

**Authors:** Tong Shu

**Affiliations:** 1 New York University Langone Health, New York, USA

In this work, the authors use a microfluidic bioreactor and quantitative phase imaging (QPI) to investigate the effect of growth-induced pressure (GIP) on intracellular dynamics in *S. cerevisiae* yeast cells. They identified a potential mechanism for the observed reduction in molecular diffusivity under confinement, attributing it to the increased dry mass density resulting from the intracellular accumulation of macromolecules. Their findings are further supported by evidence of proportional scaling between cell content and growth-induced pressure. The research is interesting and sound; however, I have a few suggestions to improve clarity and strengthen the study’s conclusions.

  

1. Please double check all references in the 2 ^nd^ version of the manuscript. For example, in the last paragraph of “Conclusion and discussion” section, the reference after “0.95M” should be #11 instead of #10. Similarly, in the first paragraph of “Bright field and fluorescent microscopy” subsection of the Method section, the reference after “work” should be #11 rather than #10, #11.
2. In the 2 ^nd^ paragraph of Method section – “Refractive index and dry mass density measurement of cells with QPI”, it is mentioned that OPD was calculated as the upper 5% of brightness within phase images of cells. Given that refractive index is calculated by dividing OPD by cell height, do these upper 5% pixels consistently align with the center of the cell, especially considering that vacuoles exhibit lower OPD values? In other words, if calculating the mean positions of these upper 5% pixels, do they align with the cell center when fitted with an ellipse?
3. In Figure 1a, two cells in the top-left corner of the OPD image lack clear boundaries. Were these cells included in the refractive index quantification? If so, will those include additional variations? If not, it will be beneficial to explicitly mention this exclusion in the Method section.
4. In the Result section of “Estimation of the nominal intracellular osmotic pressure”, should the linear extrapolation of zero dry mass density correspond to the refractive index of the media (n _media_ = 1.336) rather than the water (n _water_ = 1.332)?
5. While GIP values are inferred through hydraulic pressure calibrations, do cells at different locations within the chamber experience the same GIP? If a pressure gradient exists (e.g., higher GIP at the boundary than at the center), the averaged OPD calculated by averaging all cells might correspond to a lower average GIP value, potentially leading to a higher slope in Figure 2d. This will lead to a lower value in the estimation of nominal intracellular osmotic pressure.
6. The increase in dry mass density under GIP is attributed to the macromolecules and osmolytes production, but could it also result from a decrease in cell volume without mass production? Addressing this point would clarify the mechanisms involved. For example, does the cell volume decreases at a slower rate than the increase in dry mass density? Or does the cell volume remain constant at higher GIP while dry mass density continues to increase?
7. Finally, some language in the introduction can be slightly adjusted for better readability: For example, in the 1 ^st^ line: “Spatial confinement can be found everywhere in **living systems**, from roots sprouting in to the porous **soil, tumors** growing in the body of an organ, all the way to microbes growing in confining biofilms.” Consider rephrasing in the 9 ^th^ line:” kingdoms of the living” to “kingdoms of **life**”.

Is the study design appropriate and does the work have academic merit?

Yes

Is the work clearly and accurately presented and does it cite the current literature?

Yes

If applicable, is the statistical analysis and its interpretation appropriate?

Yes

Are all the source data underlying the results available to ensure full reproducibility?

Yes

Are the conclusions drawn adequately supported by the results?

Partly

Are sufficient details of methods and analysis provided to allow replication by others?

Partly

Reviewer Expertise:

Biophysics and cellular physiology, focusing on the interplay between the complex intracellular environment and cellular processes.

I confirm that I have read this submission and believe that I have an appropriate level of expertise to confirm that it is of an acceptable scientific standard, however I have significant reservations, as outlined above.

# Reviewer report: Reviewer response for version 2

**DOI:** 10.21956/openreseurope.20720.r48876

**Published:** 2024-12-24

**Authors:** Marco Eigenfeld

**Affiliations:** 1 Medical University of Graz, Graz, Austria

I would like to thank the authors for addressing my previous comments and making the necessary revisions. I only have a few minor remarks:

1. The authors now correctly introduce the abbreviation for *S. cerevisiae*. However, the phrase "for short" does not need to be italicized, and it can also be omitted entirely since it is already clear that this is an abbreviation.
2. The section titled **"**Cell culture and strains" could be improved. Since the study involves yeast, the term "cell culture" is not the most accurate. A more appropriate header would be "Strains and Maintenance".
3. In the section "Estimation of the nominal intracellular osmotic pressure", the formulas are nicely presented using a formula editor. However, in the section "Refractive index and dry mass density measurement of cells with QPI", the formulas are not as visually clear. I recommend formatting these formulas similarly to ensure consistency.

Apart from these minor remarks, I have no further concerns regarding the indexing, as all content-related comments have been revised appropriately.

Is the study design appropriate and does the work have academic merit?

Yes

Is the work clearly and accurately presented and does it cite the current literature?

Yes

If applicable, is the statistical analysis and its interpretation appropriate?

Yes

Are all the source data underlying the results available to ensure full reproducibility?

Partly

Are the conclusions drawn adequately supported by the results?

Yes

Are sufficient details of methods and analysis provided to allow replication by others?

Partly

Reviewer Expertise:

Investigating the mechanisms of yeast aging and yeast physiology, with a focus on the interplay between aging and protein synthesis.

I confirm that I have read this submission and believe that I have an appropriate level of expertise to confirm that it is of an acceptable scientific standard.

# Reviewer report: Reviewer response for version 1

**DOI:** 10.21956/openreseurope.20066.r45368

**Published:** 2024-11-07

**Authors:** Anahit Shirvanyan

**Affiliations:** 1 Biochemistry, Microbiology and Biotechnology, Yerevan State University, Yerevan, Armenia

The manuscript presents a detailed investigation into how growth-induced pressure (GIP) affects the intracellular dry mass density of *Saccharomyces cerevisiae* using quantitative phase imaging (QPI). The study effectively connects these physiological changes to the physical phenomena of macromolecular crowding within confined spaces. However, there are some points which I want to raise before considering accepting the manuscript. With revisions, this manuscript would be a strong addition to the field. Below are my comments:

1. Simplify Language for a Broader Audience: The manuscript is somewhat difficult for first-time readers. Some sentences are long and complex, which may hinder comprehension. Breaking these into smaller, more digestible parts would improve readability.
2. The following sentence in the introduction: ’We observed that confined growth was associated with increased dry mass density. We measured the concentration of a fluorophore inside the cell and observed that the increase in RI was proportional to the increase in fluorophore concentration. A linear extrapolation between GIP and RI down to the refractive index of water predicted the nominal intracellular osmotic pressure of the cell.’ I recommend moving the sentence to the Results section to maintain clarity and flow.
3. The introduction clearly describes GIP and its relation to the refractive index (RI) of the cell’s dry mass, as well as the novelty of the study. However, I suggest highlighting the potential applications or implications of this study to give readers a clearer understanding of its broader significance.
4. The authors mention several other techniques (e.g., SMR, cryoelectron tomography) but don’t provide much detail on why they are inadequate for their purposes. A sentence or two explaining the limitations of these methods in the context of this study would make the argument for QPI even stronger. The last sentence linking RI and osmotic pressure is a nice touch but could use a bit more explanation. For example, why is this important? How does it support the hypothesis or lead to further questions for investigation? A more explicit link between this finding and the larger context of GIP would strengthen the study.
5. In the Methods section, *S. cerevisiae strains* are mentioned, but the specific strains used are not indicated. This should be clarified.
6. If possible, I would suggest adding a diagram or image of the designed PDMS microfluidic device (chamber). This would help readers better understand the method and experimental setup.
7. Were the cells pre-inoculated in liquid media? If the inoculation was done directly from plates, it could elongate the lag phase, and some cells may be inhibited or even die. What was the initial cell concentration? This should be clarified.
8. The manuscript lacks citations for some of the methods used. If the methods are newly designed and described for the first time, this should be explicitly mentioned. If previously used methods are referenced, they should be cited.
9. In the Methods section, the phrase "Next, a single colony was inoculated in a fresh liquid SCD liquid media and incubated with orbital shaking at 200 rpm at 30°C overnight." contains a repetition of the word "liquid."
10. The manuscript uses many abbreviations. It would be helpful to include an abbreviation list for readers’ reference.
11. Some abbreviations, such as PDMS and GFP, are not defined in the text. These should be written out the first time they appear in the manuscript.
12. The sentence "To measure GFP accumulation, cytosolic GFP expressed from HIS3 promoter" requires clarification. How was GFP expressed? Does this mean the authors used genetic engineering? If so, could this influence the growth properties of the strain? If genetic manipulation was used, the methods and plasmids should be described or referenced in the Methods section.
13. The Results section contains some description of the experimental setup, which should be transferred to the Materials and Methods section. Keeping the Results focused on data interpretation would help readers engage more easily with the study’s findings.
14. Since the cells are growing in confined spaces, aeration of the medium is likely low. This could shift yeast metabolism towards fermentation, which may produce gases and increase internal pressure. Was the outlet for these gases considered in the setup? How was this accounted for in the study?
15. Can the results obtained with SCD medium be generalized to other media, such as YPD? Do the growth characteristics depend on the medium used?
16. It would be helpful to present growth differences between confined and non-confined conditions. This could help readers understand how GIP influences the growth properties of the cells.
17. If intracellular osmotic pressure rises, water from the surrounding medium would enter the cells to balance the osmotic pressure. This would likely lead to cell swelling, which could influence the optical path difference (OPD). Was this taken into consideration?
18. In the abstract, the sentence: “the observed decrease in diffusivity can be at least attributed to the intracellular accumulation of macromolecules” could be more assertive. If the authors have evidence supporting macromolecule accumulation as the primary cause, this could be emphasized more strongly.
19. The authors briefly mention that statistical analysis was performed using Python with Statsmodels, but there is no further description of the specific tests or methods used. It would be helpful to clarify which statistical tests were applied.
20. The explanation of GFP imaging and processing in ImageJ is clear, but it would help to mention how the authors handle potential variations between cells (e.g., differences in GFP expression or autofluorescence). Are the cells normalized in some way? Is there any quality control on the imaging process?
21. The results mention several figures (e.g., Figures 1a, 2a, 2d), but the description of the figures themselves is minimal. While it’s clear that the figures are central to understanding the results, it might be helpful to briefly describe the key observations or trends in each figure within the text. For example, “Figure 2b shows a clear linear decrease in OPD with increasing GIP...” This makes it easier for readers to follow without needing to cross-reference the figures repeatedly.
22. While the authors mention that the nominal osmotic pressure you estimated (1.55 MPa) is slightly higher than the 0.95 MPa from the previous model, they don’t elaborate much on why there’s a difference. Is this difference due to the experimental method you used (QPI) compared to the theoretical assumptions in the previous study? Are there any potential sources of error or variation in these measurements?

Is the study design appropriate and does the work have academic merit?

Yes

Is the work clearly and accurately presented and does it cite the current literature?

Partly

If applicable, is the statistical analysis and its interpretation appropriate?

Partly

Are all the source data underlying the results available to ensure full reproducibility?

Yes

Are the conclusions drawn adequately supported by the results?

Partly

Are sufficient details of methods and analysis provided to allow replication by others?

Partly

Reviewer Expertise:

Yeast Stress Biology, Biochemistry, Biophysics, Physiology, Bioenergetics

I confirm that I have read this submission and believe that I have an appropriate level of expertise to confirm that it is of an acceptable scientific standard, however I have significant reservations, as outlined above.

# Response

**Published:** 2024-12-09

**Authors:** Morgan Delarue

We thank Dr Shirvanyan for their critical reading of our manuscript, as well as their positive assessment of our work. Below is a point-by-point respond to the different points.  

 The manuscript presents a detailed investigation into how growth-induced pressure (GIP) affects the intracellular dry mass density of *Saccharomyces cerevisiae* using quantitative phase imaging (QPI). The study effectively connects these physiological changes to the physical phenomena of macromolecular crowding within confined spaces. However, there are some points which I want to raise before considering accepting the manuscript. With revisions, this manuscript would be a strong addition to the field. Below are my comments:

1. Simplify Language for a Broader Audience: The manuscript is somewhat difficult for first-time readers. Some sentences are long and complex, which may hinder comprehension. Breaking these into smaller, more digestible parts would improve readability.

**Response: **We rephrased where necessary to keep sentences short and simplified some parts, improving readability.  

1. The following sentence in the introduction: ’We observed that confined growth was associated with increased dry mass density. We measured the concentration of a fluorophore inside the cell and observed that the increase in RI was proportional to the increase in fluorophore concentration. A linear extrapolation between GIP and RI down to the refractive index of water predicted the nominal intracellular osmotic pressure of the cell.’ I recommend moving the sentence to the Results section to maintain clarity and flow.

**Response: **We deleted this part of the introduction to slightly rephrased the last sentence, in order to only introduce the results and not presenting them.  

1. The introduction clearly describes GIP and its relation to the refractive index (RI) of the cell’s dry mass, as well as the novelty of the study. However, I suggest highlighting the potential applications or implications of this study to give readers a clearer understanding of its broader significance.

**Response: **We agree with the reviewer about the importance of these applications. We wrote two sentences about the significance of regulation of mass and growth. However, we did not include this part in the introduction but in the discussion, as this read more like a discussion.  

1. The authors mention several other techniques (e.g., SMR, cryoelectron tomography) but don’t provide much detail on why they are inadequate for their purposes. A sentence or two explaining the limitations of these methods in the context of this study would make the argument for QPI even stronger. The last sentence linking RI and osmotic pressure is a nice touch but could use a bit more explanation. For example, why is this important? How does it support the hypothesis or lead to further questions for investigation? A more explicit link between this finding and the larger context of GIP would strengthen the study.

**Response: **We now explain in the introduction that the fact that we cannot recover the cells from the system limits the type of measurements we can perform. Since we took out the part related to the results, the sentence linking osmotic pressure to RI does not need more explanation.  

1. In the Methods section, *S. cerevisiae strains* are mentioned, but the specific strains used are not indicated. This should be clarified.

**Response: **This has been clarified in the revised version.  

1. If possible, I would suggest adding a diagram or image of the designed PDMS microfluidic device (chamber). This would help readers better understand the method and experimental setup.

**Response: **We amended Figure 2a to add descriptions on it, clarifying how it is used in terms of pressure measurement and feeding of the cells.  

1. Were the cells pre-inoculated in liquid media? If the inoculation was done directly from plates, it could elongate the lag phase, and some cells may be inhibited or even die. What was the initial cell concentration? This should be clarified.

**Response: **All this was already clarified in the first section of the Methods part.  

1. The manuscript lacks citations for some of the methods used. If the methods are newly designed and described for the first time, this should be explicitly mentioned. If previously used methods are referenced, they should be cited.

**Response: **We added one citation to our study to confine cells, but do not really see which specific methods the Reviewer is mentioning. There is no specific method which stands as non-conventional, besides QPI which is deeply explained, in our opinion. If we are missing some particular point, we would be grateful for this Reviewer to elaborate.  

1. In the Methods section, the phrase "Next, a single colony was inoculated in a fresh liquid SCD liquid media and incubated with orbital shaking at 200 rpm at 30°C overnight." contains a repetition of the word "liquid."

**Response: **We corrected this – and point to the fact that this is the response to your query #7.  

1. The manuscript uses many abbreviations. It would be helpful to include an abbreviation list for readers’ reference.

**Response: **We added an abbreviation section, after the discussion.  

1. Some abbreviations, such as PDMS and GFP, are not defined in the text. These should be written out the first time they appear in the manuscript.

**Response: **This has been corrected in the method section of the paper.  

1. The sentence "To measure GFP accumulation, cytosolic GFP expressed from HIS3 promoter" requires clarification. How was GFP expressed? Does this mean the authors used genetic engineering? If so, could this influence the growth properties of the strain? If genetic manipulation was used, the methods and plasmids should be described or referenced in the Methods section.

**Response: **We further detailed the genetic construct of our strain, which was a gift, and precise that its growth rate is unaffected.  

1. The Results section contains some description of the experimental setup, which should be transferred to the Materials and Methods section. Keeping the Results focused on data interpretation would help readers engage more easily with the study’s findings.

**Response: **We moved some microfluidic method part from the results section to the methods section.  

1. Since the cells are growing in confined spaces, aeration of the medium is likely low. This could shift yeast metabolism towards fermentation, which may produce gases and increase internal pressure. Was the outlet for these gases considered in the setup? How was this accounted for in the study?

**Response: **PDMS is permeable to gases, and the chamber is thin, such that the cells have an equal amount of oxygen, which is renewed through diffusion in the material. Similarly, because it is permeable, gases, if any, will not accumulate in the culture chamber. Thus, gases are not an issue in our setup, both in the case of metabolic activity as well as in the pressure measurement. In any cases, if gas was to be produced faster than it could diffuse out of the chamber through PDMS, we would clearly see it as the refractive index would be much lower than the one of the liquid.  

1. Can the results obtained with SCD medium be generalized to other media, such as YPD? Do the growth characteristics depend on the medium used?

**Response: **YPD is a richer and less controlled medium (in terms of its composition) than SCD, which is slightly auto-fluorescent thus limiting imaging in some cases (imaging of the GEMs nanoparticles for instance – Alric et al, Nat Phys, 2022). These disadvantages have led us to prefer SCD as a reference culture medium for yeast studies. In YPD, cells tend to have a slightly faster basal proliferation rate. However, there are no reasons for the generic results that we obtained to be dependent on the culture medium: under confinement, any cells and organisms we have investigated so far show a decreased growth concomitant with a higher crowding. This is true for yeasts (Alric et al, Nat Phys, 2022), but also for mammalian cells (Ben Meriem et al, Lab Chip 2023) and bacteria (Leblanc et al, bioRxiv, 2024). It is also true for other types of yeasts or bacteria (unpublished results). It could indeed be that the magnitude of this effect depends on the culture conditions (richness of the medium, but also osmolarity, pH, etc) because the cells could have different basal biophysical properties. Although interesting, the investigation of such effects, linking metabolism to cellular biophysical properties, goes far beyond the scope of this article.  

1. It would be helpful to present growth differences between confined and non-confined conditions. This could help readers understand how GIP influences the growth properties of the cells.

**Response: **We did not add such data because they are already published, and the publications are listed in the introduction (1-10). For yeasts, in particular, it has already been published in Delarue et al, Nat Phys, 2016 and in Alric et al, Nat Phys, 2022. We do not think adding already-known results to the manuscript would increase its readability.  

1. If intracellular osmotic pressure rises, water from the surrounding medium would enter the cells to balance the osmotic pressure. This would likely lead to cell swelling, which could influence the optical path difference (OPD). Was this taken into consideration?

**Response: **Indeed, if cells were freely growing, a rise in intracellular osmotic pressure with regard to the external would lead to water influx and cell swelling, just like a hypo-osmotic stress. In our situation, the rise in intracellular pressure is mechanically balanced by the surroundings: the increased pressure is deforming surrounding cells and confining chamber. In our case, we measured that cell volume does not change (neither increase nor decrease, see Alric et al, Nat Phys, 2022). When we relax the confinement, cells do swell, because they have space to do so and are not in a mechanical equilibrium anymore. Crowding does decrease, with a decrease in intracellular diffusion of the GEMs nanoparticles. In any case, if a cell was to get diluted by water influx, this would decrease crowding and intracellular density, and thus decrease OPD. There is nothing to consider in the sense that this would be measured, and we would observe a lower-than-expected OPD. As such, no correction is needed.  

1. In the abstract, the sentence: “the observed decrease in diffusivity can be at least attributed to the intracellular accumulation of macromolecules” could be more assertive. If the authors have evidence supporting macromolecule accumulation as the primary cause, this could be emphasized more strongly.

**Response: **We replaced “can be at least” by “could be”. We still cannot be 100% assertive, as we have not measured other parameters, such as pH, which could also influence the motion of the nanoparticles.   

1. The authors briefly mention that statistical analysis was performed using Python with Statsmodels, but there is no further description of the specific tests or methods used. It would be helpful to clarify which statistical tests were applied.

**Response: **We added more details about the statistical functions we used in the Methods section.  

1. The explanation of GFP imaging and processing in ImageJ is clear, but it would help to mention how the authors handle potential variations between cells (e.g., differences in GFP expression or autofluorescence). Are the cells normalized in some way? Is there any quality control on the imaging process?

**Response: **We measured in control cells the autofluorescence (as was mentioned in the method section) and the surrounding fluorescent background, and subtracted both from the signal. Moreover, the fluorescence intensity was homogenous, suggesting that there were no large variations in between cells. We added comments on these aspects in the method section.  

1. The results mention several figures (e.g., Figures 1a, 2a, 2d), but the description of the figures themselves is minimal. While it’s clear that the figures are central to understanding the results, it might be helpful to briefly describe the key observations or trends in each figure within the text. For example, “Figure 2b shows a clear linear decrease in OPD with increasing GIP...” This makes it easier for readers to follow without needing to cross-reference the figures repeatedly.

**Response: **We rephrased where needed the description of the different sub-figures.  

1. While the authors mention that the nominal osmotic pressure you estimated (1.55 MPa) is slightly higher than the 0.95 MPa from the previous model, they don’t elaborate much on why there’s a difference. Is this difference due to the experimental method you used (QPI) compared to the theoretical assumptions in the previous study? Are there any potential sources of error or variation in these measurements?

**Response: **We added a small discussion at the end of the manuscript, to explain this potential discrepancy. Indeed, we assume that the theoretical assumptions in the previous study could be oversimplified, and would tend to trust this estimation more. So far, we have no means of testing the previous hypothesis independently.

# Reviewer report: Reviewer response for version 1

**DOI:** 10.21956/openreseurope.20066.r45367

**Published:** 2024-11-06

**Authors:** Marco Eigenfeld

**Affiliations:** 1 Medical University of Graz, Graz, Austria

Summary of the main findings:

 The authors effectively demonstrate the link between dry cell density, as measured through refractive index changes, and the stress induced by growth in *Saccharomyces* yeast cells. The findings observed in normal yeast strains are further validated by incorporating GFP-expressing yeast, which supports the study's primary objectives.

 Recommendations to

 Introduction:

 The introduction would benefit from an initial list of confined spaces, which would set the context more effectively. I suggest removing the brackets and abbreviation "SMR" since it is not reused elsewhere in the text. Additionally, please introduce *S. cerevisiae* by following its full name with the abbreviation in parentheses for clarity. In the third section of the introduction, the full name is used again, as well as in the methods section; please use the abbreviation consistently throughout.

 At the end of the first section, the authors refer to “other effects” in plural, but only mention a single effect. Either change this to singular or provide an additional example to maintain consistency. A transition between the first and second sections would help improve the flow, as the text currently shifts abruptly from cytoplasmic fluidity effects to dry mass density.

 If "numerous methods" are mentioned in plural, please list some specific methods along with references to avoid a potentially unsupported assertion. Currently, these methods are discussed following an unrelated sentence, “To develop GIP, cells are proliferating inside a confining microfluidic chamber and become densely packed,” which could disrupt readability.

 M&M section:

 In the methodology, please add the flow speed at which the cell suspension was introduced via syringe for completeness. Additionally, correct the typo “inversemicroscope” in the microscopy section. Clarify how many cells were measured for the refractive index (RI) and microscopy; this information would be helpful for assessing the robustness of the data.

 The inclusion of GFP expression measurement is commendable. However, details regarding the expression mechanism and yeast strain used are currently missing. Please incorporate a brief paragraph in the methods section that specifies the strain and describes how GFP expression is regulated.

 Results:

 On page 4, revising the last section for readability would be beneficial. Presently, nearly every sentence begins with “we,” and introducing variation here would improve style and readability. On page 5, there is an incomplete sentence: “Importantly, intracellular density extrapolated to 0 when the fluorescence intensity did,” which requires revision for clarity.

 Discussion:

 Lastly, there appears to be some mixing of results and discussion in the section “Refractive index and dry mass density of *S. cerevisiae* determined with QPI.” In this section, the authors contextualize their findings within existing literature, which would typically suit a discussion section. However, the later sections only describe findings, fitting the results format. To enhance consistency, I suggest either maintaining a combined results and discussion structure or introducing a distinct discussion section to compare results with literature values. The second paragraph of the conclusion also seems more aligned with a discussion, and moving this content could strengthen both sections.

Is the study design appropriate and does the work have academic merit?

Yes

Is the work clearly and accurately presented and does it cite the current literature?

Yes

If applicable, is the statistical analysis and its interpretation appropriate?

Yes

Are all the source data underlying the results available to ensure full reproducibility?

Partly

Are the conclusions drawn adequately supported by the results?

Yes

Are sufficient details of methods and analysis provided to allow replication by others?

Partly

Reviewer Expertise:

Investigating the mechanisms of yeast aging and yeast physiology, with a focus on the interplay between aging and protein synthesis.

I confirm that I have read this submission and believe that I have an appropriate level of expertise to confirm that it is of an acceptable scientific standard, however I have significant reservations, as outlined above.

# Response

**Published:** 2024-12-09

**Authors:** Morgan Delarue

We thank Dr Eignefeld for their critical reading of our manuscript, as well as their positive assessment of our work. Below is a point-by-point respond to the different points.   Summary of the main findings:

 The authors effectively demonstrate the link between dry cell density, as measured through refractive index changes, and the stress induced by growth in *Saccharomyces* yeast cells. The findings observed in normal yeast strains are further validated by incorporating GFP-expressing yeast, which supports the study's primary objectives.

 Recommendations to

 Introduction:

 The introduction would benefit from an initial list of confined spaces, which would set the context more effectively. We added two sentences to list some cases of confinement.   I suggest removing the brackets and abbreviation "SMR" since it is not reused elsewhere in the text. Agreed and done.   Additionally, please introduce *S. cerevisiae* by following its full name with the abbreviation in parentheses for clarity. In the third section of the introduction, the full name is used again, as well as in the methods section; please use the abbreviation consistently throughout.

**Response:** Agreed and done.

 At the end of the first section, the authors refer to “other effects” in plural, but only mention a single effect. Either change this to singular or provide an additional example to maintain consistency. Indeed, thank you for noting. We added the case of pH which by changing electrostatic interactions can play on the effective viscosity of the cytoplasm.   A transition between the first and second sections would help improve the flow, as the text currently shifts abruptly from cytoplasmic fluidity effects to dry mass density. We reformulated the end of the first section to finish with dry mass density.

 If "numerous methods" are mentioned in plural, please list some specific methods along with references to avoid a potentially unsupported assertion. Currently, these methods are discussed following an unrelated sentence, “To develop GIP, cells are proliferating inside a confining microfluidic chamber and become densely packed,” which could disrupt readability. **Response:** We rephrased this part.

 M&M section:

 In the methodology, please add the flow speed at which the cell suspension was introduced via syringe for completeness. We inject them by hands, and do not have this measurement. We added a sentence giving orders of magnitude of cell number and timing to be more descriptive.   Additionally, correct the typo “inversemicroscope” in the microscopy section. Done   Clarify how many cells were measured for the refractive index (RI) and microscopy; this information would be helpful for assessing the robustness of the data. We clarified the statistics in the captions of the figures.

 The inclusion of GFP expression measurement is commendable. However, details regarding the expression mechanism and yeast strain used are currently missing. Please incorporate a brief paragraph in the methods section that specifies the strain and describes how GFP expression is regulated. We added a description in the “Cell culture conditions” part, which was renamed “Cell culture conditions and strain”.  

 Results:

 On page 4, revising the last section for readability would be beneficial. Presently, nearly every sentence begins with “we,” and introducing variation here would improve style and readability. We rephrased this section.   On page 5, there is an incomplete sentence: “Importantly, intracellular density extrapolated to 0 when the fluorescence intensity did,” which requires revision for clarity.

**Response:** The sentence was not incomplete, but was indeed not clearly written. We rephrased for clarity.

 Discussion:

 Lastly, there appears to be some mixing of results and discussion in the section “Refractive index and dry mass density of *S. cerevisiae* determined with QPI.” In this section, the authors contextualize their findings within existing literature, which would typically suit a discussion section. However, the later sections only describe findings, fitting the results format. To enhance consistency, I suggest either maintaining a combined results and discussion structure or introducing a distinct discussion section to compare results with literature values. The second paragraph of the conclusion also seems more aligned with a discussion, and moving this content could strengthen both sections.

**Response: **We moved the result part to the conclusion, which we renamed “Conclusion and discussion”, to increased readability.
